# Mechanisms for the Cognitive Processing of Attractiveness in Adult and Infant Faces: From the Evolutionary Perspective

**DOI:** 10.3389/fpsyg.2020.00436

**Published:** 2020-03-11

**Authors:** Hui Kou, Qinhong Xie, Taiyong Bi

**Affiliations:** ^1^Center for Mental Health Research in School of Management, Zunyi Medical University, Guizhou, China; ^2^School of Criminal Justice, China University of Political Science and Law, Beijing, China

**Keywords:** adult face, infant face, facial attractiveness, cognitive processing, evolutionary psychology

## Abstract

Research on the cognitive processing of facial attractiveness has mainly focused on adult faces. Recent studies have revealed that the cognitive processing of facial attractiveness in infant faces is not the same as that in adult faces. Therefore, it is necessary to summarize the evidence on the processing of facial attractiveness in each kind of face and compare their underlying mechanisms. In this paper, we first reviewed studies on the cognitive processing of facial attractiveness in adult faces, including attentional and mnemonic processing, and then discussed the underlying mechanisms. Afterward, studies on facial attractiveness in infant faces were reviewed, and the underlying mechanisms were also discussed. Direct comparisons between the two kinds of cognitive processing were subsequently made. The results showed that the mechanisms for the processing of attractiveness in adult faces and infant faces are mainly motivated by the perspectives of mate selection and raising offspring, respectively, in evolutionary psychology. Finally, directions for future research are proposed.

## Introduction

Faces can be automatically evaluated on multiple trait dimensions. Through a dimensional reduction technique, varieties of traits of adult (Oosterhof and Todorov, 2008; Sutherland et al., 2013) and infant faces (Collova et al., 2019) were reduced to “valence/approachability” for adult faces and “niceness” for infant faces respectively. Facial attractiveness, which refers to the pleasant emotional experience and approaching intention induced by a human face (Rhodes, 2006), is closely associated to the general positivity/negativity in evaluations of adult and infant faces. Facial attractiveness is also proposed to be divided into two components: sexual attractiveness and cuteness (Rhodes, 2006). Later, Geldart (2010) proposed that female beauty can be mainly described by attractiveness, prettiness, beauty and cuteness. Evidently, attractiveness in adult faces may be mainly characterized by prettiness and beauty, while that in infant faces may be mainly characterized by cuteness. Evidence has shown that the evaluation of cuteness in a face is independent of that of attractiveness, prettiness and beauty (Geldart, 2010). Thus, there may be different mechanisms for the cognitive processing of attractiveness in adult faces and infant faces. However, studies on facial attractiveness mainly focus on adult faces. In the present review, we summarized and compared the cognitive processing of facial attractiveness in both adult and infant faces. Different and common underlying mechanisms were then discussed to provide a more comprehensive view of facial attractiveness.

## Cognitive Processing of Facial Attractiveness in Adult Faces

Studies on the cognitive processing of facial attractiveness in adult faces have mainly focused on attentional processing and mnemonic processing. The results are usually interpreted based on the perspective of mate selection in evolutionary psychology.

Highly attractive (HA) faces can attract visual attention (Sui and Liu, 2009; Lindell and Lindell, 2014), which is evident in newborns and infants (Slater et al., 2000; Van Duuren et al., 2003; Griffey and Little, 2014). Moreover, facial attractiveness may influence visual attention in several ways. Please note that the level of attentional bias induced by facial attractiveness may be different between men and women. We may discuss the influence of observers’ sex in later paragraphs. In this paragraph, we only focus on the attentional bias induced by facial attractiveness regardless of observers’ sex. First, compared with lowly attractive (LA) faces, individuals showed attentional maintenance toward and attentional disengagement from HA faces (Maner et al., 2007a, b; Silva et al., 2016). For example, studies of eye-movement tracking revealed that individuals showed a longer fixation duration on HA faces than that on LA faces (Shimojo et al., 2003; Dewall and Maner, 2008; Kou et al., 2016), which were also evident when faces were displayed in natural scenes (Leder et al., 2010, 2016; Mitrovic et al., 2016). Second, target-tracking behavior or the sustained attention can be modulated by the attractiveness of the target and distractor. For example, a recent study showed higher performance for the tracking of HA faces than for that of LA faces when participants were required to track moving faces among distractors and report the final location of each target (Li et al., 2016). When HA faces were distractors, the success rate for target tracking was reduced (Liu and Chen, 2012). Facial attractiveness influences attention-related neural activities, such as late positive potential (LPP) which is a late positive component in event-related potential (ERP) waves. One of the potential functions of LPP might be indicating the attentional allocation. From this perspective, researchers found that HA cartoon faces induced a larger LPP than did LA faces (Lu et al., 2014). However, it should be noted that the function of LPP is complex. Whether other functions played important roles in these results should be further investigated.

Why do people pay more attention to HA faces? The perspective of mate selection in evolutionary psychology may provide answers to this question. According to mate selection theory, women may emphasize the social position of potential mates, while men may focus on the attractiveness of potential mates (Dewall and Maner, 2008). In accordance with this theory, studies have shown that the attentional bias to HA faces is strongly affected by biological factors such as the sex of the targets and the hormone level of the observers.

First, the sex of the targets may determine the effect of facial attractiveness on visual attention. Some studies have revealed that men may show a stronger attentional bias toward HA opposite-sex faces than women may (Fugita et al., 1977; van Straaten et al., 2010; Valuch et al., 2015; Mitrovic et al., 2018). For example, evidence showed that heterosexual men spent less time fixating on LA faces than on HA opposite-sex faces, while there was no significant difference of fixation time between LA and HA faces among heterosexual women (van Straaten et al., 2010). Neurophysiological evidence also revealed that HA opposite-sex faces elicited larger N170 and LPP than LA opposite-sex faces did for heterosexual men (Morgan and Kisley, 2014; Zhang et al., 2018). Further studies have shown that both female and male participants demonstrate attentional bias toward HA female faces but not toward HA male faces (Maner et al., 2003, 2007a). For example, an eye-movement tracking study revealed that participants showed longer total fixation time and first fixation time for HA faces compared with LA faces. Notably, the difference of fixation time between HA and LA faces was larger for female faces than that for male faces (Leder et al., 2010). Consistently, neurophysiological evidence has also suggested that heterosexual women showed a greater difference in LPP response to the HA versus LA same-sex faces than did heterosexual men (Hahn et al., 2016). According to evolutionary theory, women may focus on their potential rivals in mate selection, as these HA rivals are threats for these women in attracting satisfactory mates (Maner et al., 2007b; Maner and Ackerman, 2015). Therefore, women may also show attentional bias to HA female faces. However, HA men may not be valid threats to other men in mate selection. Men may thus not show attentional bias toward HA male faces. Therefore, evolutionary theory predicts that both men and women will show attentional bias toward HA female faces.

Second, the romantic relationship status and hormone level of the observer may also play an important role in determining his/her attentional bias toward HA faces. According to evolutionary theory, it can be hypothesized that compared with non-single individuals, single individuals may allocate more attentional resources to HA individuals. Consistently, studies have revealed less attentional bias toward HA faces among participants who were primed by romantic love or in a committed romantic relationship (Maner et al., 2008; Itier and Batty, 2009). Comparatively, there was stronger correlation between total fixation duration and facial attractiveness in single participants (Leder et al., 2016). Importantly, a longitudinal study found that heterosexual individuals pay less attention to HA faces after building romantic relationships (Koranyi and Rothermund, 2012).

Lastly, evolutionary theory also predicts that women in a period of fertility may pay more attention to HA male faces, as such faces may be considered representative of high genetic quality. Consistently, women near ovulation with high fertility spent more time on looking at HA male faces than women with low fertility did (Anderson et al., 2010). Electrophysiological evidence has also indicated that the visual mismatch negativity (vMMN) induced by HA male faces is greater among heterosexual women in the ovulatory phase than among those in the menstrual phase (Zhang et al., 2018). This vMMN is an indicator of automatic attentional processing (Kremláček et al., 2016). Taken together, these findings suggest that biological factors significantly influence attentional bias toward HA faces and support evolutionary theory.

Similar to attentional processing, facial attractiveness may also influence mnemonic processing. However, the effects of facial attractiveness on mnemonic processing are inconsistent. For example, previous studies have found that the memory of HA faces is superior to that of LA faces among heterosexual individuals (Zhang et al., 2016), while another study have found opposite results (Wiese H. et al., 2014). Neuroimaging evidence showed that better memory for HA faces reflects stronger functional connectivity between reward-related orbito-frontal cortex (OFC) and memory-related hippocampal regions (Itier and Batty, 2009). Moreover, studies have found that the memory of both HA and LA faces is better than that of moderately attractive faces (Fleishman et al., 1976; Lin et al., 2016). These inconsistent results suggest that the mnemonic processing of facial attractiveness may be affected by complex factors. Nevertheless, evolutionary theory is still the basic theoretical framework for explaining the mnemonic processing of facial attractiveness.

Consistent with evolutionary theory, biological factors, such as the sex of targets and the hormone level of observers, also play important roles in the mnemonic processing of facial attractiveness. First, HA female faces are remembered better than are HA male faces. For example, a study concerning spatial episodic memory revealed that both men and women show better performance in memorizing the locations of HA female faces but not male faces (Becker et al., 2005). Correspondingly, an ERP study revealed higher neural activities in the learning and recognition stages for HA female faces than for LA female faces among heterosexual individuals (Zhang et al., 2011). All of this evidence suggests that the memory of HA female faces is better than that of LA female faces. Second, the mnemonic processing of facial attractiveness is affected by romantic relationship status. According to evolutionary psychology theory, it can be hypothesized that women who are in love and in stable relationships may have poor memory of HA male faces, which is beneficial for the stability of their romantic relationships. Consistently, women involved in a heterosexual romantic relationship perform worse in memorizing HA male faces than do single women (Karremans et al., 2011). Moreover, heterosexual women in high-quality romantic relationships have more false memories of HA male faces (Watkins et al., 2017).

Unlike attentional processing, the mnemonic processing of facial attractiveness is affected by additional factors. The most important factor is facial characteristics. In addition to facial attractiveness, facial distinctiveness may also affect mnemonic processing (Sarno and Alley, 1997; Wickham and Morris, 2003; Bainbridge et al., 2013). Facial distinctiveness refers to the extent that a face deviates from the average face. Faces with greater distinctiveness have been found to be remembered better (Sarno and Alley, 1997). Although some studies have indicated that attractiveness has a monotonic relationship with distinctiveness, others have suggested that this relationship is U-shaped. For example, some studies have found that facial attractiveness is negatively correlated with facial distinctiveness (Rhodes and Tremewan, 1996; Peskin and Newell, 2004; Rhodes, 2006), indicating lower distinctiveness for HA faces. In contrast, other studies have found that atypical faces (both HA and LA faces) are more distinctive than are average faces (Perrett et al., 1994; Wickham and Morris, 2003; Mende-Siedlecki et al., 2013). Obviously, a more distinctive face is easier to remember than a less distinctive one. Therefore, mixed results have been found in studies concerning the mnemonic processing of facial attractiveness, without controlling for the factor of distinctiveness.

In summary, existing evidence from behavioral, eye-movement and electrophysiological studies reveal the effect of facial attractiveness in adult faces on the attentional and mnemonic processing. However, only a small number of fMRI studies are designed to investigate the neural mechanisms underlying the attentional and mnemonic processing of facial attractiveness. Usually, a HA face is considered an incentive which may activate the reward circuit involving the nucleus accumbens (NAcc), medial prefrontal, dorsal anterior cingulate and OFC (Senju and Csibra, 2008). An fMRI study found that the activations in OFC were different between HA and LA opposite-sex faces only for men, which was in accordance with the evolutionary theory (Cloutier et al., 2008). Furthermore, researchers investigate how task and attentional state influence the brain activities induced by attractiveness. First, passive viewing HA female faces may activate the NAcc, while subcortical and paralimbic reward regions are activated only when participants were performing an active keypress task (Aharon et al., 2001). Second, brain activities in the ventral striatum were found to increase with facial attractiveness, only when the eye gaze of the face was direct (Kampe et al., 2001). Third, when participants judged facial attractiveness explicitly, neural activities in a widely distributed network involving the ventral occipital, anterior insular, dorsal posterior parietal, inferior dorsolateral, and medial prefrontal cortices were correlated with the level of facial attractiveness, while such associations only remained in ventral occipital region when subjects were not attending explicitly to facial attractiveness (Gredebäck et al., 2010). These fMRI results support the important role of reward circuit in active processing of facial attractiveness. However, it needs further studies to investigate how facial attractiveness biases attention and memory.

## Cognitive Processing of Facial Attractiveness in Infant Faces

The human infant face is a visual stimulus with evolutionary significance. The preference for infant faces is cross-culturally consistent (Esposito et al., 2014). Studies have shown that individuals tend to pay attention to infant faces (Tobias et al., 2007; Lucion et al., 2017). Unlike facial attractiveness in adult faces, the evolutionary value of facial attractiveness in infant faces is not mate selection. The findings concerning infant faces are usually interpreted in evolutionary psychology from the perspective of raising offspring.

As we previously noted, sex attractiveness and cuteness are two main characteristics of facial attractiveness in adult and infant faces, respectively. Facial cuteness is a facial trait associated with infants and toddlers, such as having large and round eyes, a large head, a round face, and chubby cheeks. These cute facial features construct the infant schema, which could promote the caretaking behavior of observers (Kringelbach et al., 2016). Infant faces with high infant schema traits have been rated as cuter than other infant faces (Borgi et al., 2014). Furthermore, cuter infant faces may elicit stronger positive emotional responses (Almanza-Sepulveda et al., 2018) and stronger caretaking motivation (Glocker et al., 2009a). An electromyography study revealed that unattractive infant faces elicited more corrugator supercilii and levator labii superioris movements which were indicators for negative affect (Bräuer et al., 2005). Therefore, facial attractiveness in infant faces is determined by the facial cuteness.

Facial attractiveness in infant faces can affect the attention and other cognitive processing of such faces. Evidence has revealed that both adults and children may spend more time watching cuter infant faces than less cute infant faces (Hildebrandt and Fitzgerald, 1978; Parsons et al., 2011a; Téglás et al., 2012; Sprengelmeyer et al., 2013; Borgi et al., 2014). Correspondingly, neurophysiological studies have shown that cute infant faces activate the brain regions associated with attention such as the precuneus when participants is asked to assess the cuteness of faces (Glocker et al., 2009b). These results indicate the observer preference for HA infant faces.

According to the perspective in evolutionary psychology of raising offspring, women play more important roles in raising offspring than do men. Therefore, evolutionary theory may predict that women care more about infant faces and are more sensitive to facial attractiveness in infant faces. Studies showed that women who were mothers smiled more frequently to infant faces, and showed larger skin conductance to their own infant (Téglás et al., 2012). Infant faces also elicited stronger and more stable attentional bias for women than for men (Posner and Cohen, 1984). As expected, evidence has further shown that women make greater efforts and spend more time looking at cute infant faces than do men, in a key-press task which allowed them to control the presenting duration of stimuli (Hahn et al., 2013). In addition, women are more sensitive to different levels of cuteness in infant faces and generally have more positive feelings toward infant faces (Glocker et al., 2009a; Sprengelmeyer et al., 2009; Lobmaier et al., 2010; Parsons et al., 2011b; Lehmann et al., 2013). At the neurophysiological level, cute infant faces activate the brain regions of unmarried women, which are related to reward and desire motivation, such as the striatum/NAcc and ventromedial prefrontal cortex (Glocker et al., 2009b; Yin et al., 2017). Taken together, these results indicate that cuter infant face might be a significant reward for women and thus enhance their cognitive processing of these faces.

Consistent with evolutionary theory, other factors related to raising offspring can also affect the cognitive processing of attractiveness in infant faces. First, the hormone level of the observer is an important factor. For example, premenopausal women are more sensitive to facial cuteness than are postmenopausal women and women using oral contraceptives that can artificially raise their hormone levels are more sensitive to facial cuteness (Sprengelmeyer et al., 2009), and women during ovulation are also more sensitive to cuteness differences than are women during the luteal phase (Al-Janabi and Finkbeiner, 2014). In addition, the viewing time of cute infant faces is longer for women whose level of saliva testosterone is high (Hahn et al., 2015a). The second factor is maternal tendencies, which are associated with interest in interacting with babies. Women with stronger maternal tendencies are more likely to press buttons to increase their viewing time of cute infant faces (Hahn et al., 2015b), which is consistent with evolutionary theory. Finally, in addition to the factors of the observers, other factors may influence the willingness to raise offspring and thus the cognitive processing of facial attractiveness in infant faces. For example, infant faces with abnormalities (e.g., cleft lip, strabismus, and hemangioma) are rated less attractive (Lewis et al., 2017; Huffmeijer et al., 2018), and less time is spent by the observers on viewing these faces compared with healthy faces (Parsons et al., 2011b). Another study has shown that mothers of infants with cleft lips allocated significantly less visual attention to their children’s faces, especially to the area around the mouth (De Pascalis et al., 2017). Correspondingly, an electrophysiological study revealed lower neural activities (N170 and P2) induced by infant faces with cleft lip compared with healthy infant faces (Huffmeijer et al., 2018).

## Comparison of the Cognitive Processing of Facial Attractiveness in Adult and Infant Faces

According to evolutionary theory, two events are crucial for human reproduction: mate selection and raising offspring. To achieve these two objectives, humans may develop abilities to process stimuli related to these objectives. Visual stimuli, especially faces, provide the main cues for interpersonal communication. Facial attractiveness may be adaptations for mate choice because it signals important aspects of mate quality, such as health, high genetic quality and fertility (Thornhill and Gangestad, 1999; Fink and Penton-Voak, 2002; Rhodes, 2006). It is thus quite natural that humans have cognitive bias toward HA faces. However, the motivations underlying the bias toward HA adult faces and HA infant faces are different. Therefore, there are differences and linkages between the mechanisms underlying the cognitive processing of facial attractiveness in adult and infant faces.

Regarding the common mechanisms, evidence has consistently shown that the cognitive processing of both adult and infant faces can be enhanced with the increase in facial attractiveness (Sui and Liu, 2009; Senese et al., 2013). At the behavioral level, attentional bias toward HA faces has been found in both adult and infant faces, indicated by response time or eye movement (Shimojo et al., 2003; Maner et al., 2007a, b; Dewall and Maner, 2008; Sui and Liu, 2009; Senese et al., 2013; Sprengelmeyer et al., 2013; Borgi et al., 2014; Kou et al., 2016; Leder et al., 2016; Mitrovic et al., 2016; Silva et al., 2016). At the neurophysiological level, HA faces may induce higher brain responses than LA faces in brain areas (e.g., striatum/NAcc, precuneus, ventromedial prefrontal cortex) or ERP components (e.g., N170, N2, early posterior negativity (EPN), LPP, vMMN) which may be related to attentional and rewarding processing (Werheid et al., 2007; Glocker et al., 2009b; van Hooff et al., 2011; Chen et al., 2012; Zhang and Deng, 2012; Morgan and Kisley, 2014; Wiese H. et al., 2014; Hahn et al., 2016). These common patterns of cognitive processing are consistent with evolutionary psychology, demonstrating the evolutionary significance of facial attractiveness in both adult and infant faces.

Other than common processing, there are differences between the processing of facial attractiveness in adult faces and that in infant faces. For example, the attentional bias toward HA faces is determined by the sex of the target and of the observer for adult faces and infant faces, respectively. As noted above, these differences may result from the different sources of reproductive motivation. The motivations for adult and infant facial processing derive from mate selection and raising offspring, respectively. As the theoretical frameworks are different, the findings regarding the cognitive processing of facial attractiveness in adult and infant faces should be discussed separately.

Regarding the adult face, evolutionary theory predicts that any faces conducive to choosing the right mate will be considered attractive. Under this theoretical framework, a hypothesis – “female beauty captures attention” – is proposed. According to this hypothesis, relative to male faces, HA female faces may be treated with more cognitive bias. There are three reasons that only female faces can capture attention. First, in mate selection, men may want to seek out high-quality potential mates to increase their possibility of producing offspring, and thus, facial attractiveness in female faces is an effective indicator. Second, although women may have the same motivation as men in mate selection, they may place more emphasis on the social resources and social status of the mate (Li et al., 2002; Dewall and Maner, 2008). Relatively, facial attractiveness in male faces is not as important for women as is facial attractiveness in female faces for men. Although women showed attention bias toward HA male faces compared with LA male faces (Silva et al., 2016), the attentional bias toward HA opposite-sex faces was significantly stronger for men than that for women (Kleck and Rubenstein, 1975; Fugita et al., 1977; van Straaten et al., 2010; Valuch et al., 2015; Mitrovic et al., 2018). Nevertheless, we should notice that the attractiveness in male faces may be different for women in different situations (e.g., in the ovulatory phase or not). Third, another motivation for women in mate selection is to find potential same-sex rivals to develop appropriate strategies to prevent their current or future partners from being attracted to these HA rivals (Maner et al., 2007b). Given that cognitive resources are limited, compared with allocating cognitive resources to HA male mates, allocating cognitive resources to competitive same-sex rivals makes more sense. Therefore, evolutionary theory predicts that only HA female faces can capture attention from both men and women, and numerous results support this hypothesis (Maner et al., 2003, 2007a; Levy et al., 2008; Lovén et al., 2011).

Although the “female beauty captures attention” hypothesis is supported by a series of studies, other evidence is in favor of another hypothesis – “opposite-sex beauty captures attention” – which proposes that both men and women will show selective attention to HA opposite-sex faces (Maner et al., 2003). For example, a study has found that women also show attention bias toward HA male faces compared with LA male faces (Silva et al., 2016). Neurophysiological also revealed that HA opposite-sex faces induced a larger LPP than did LA faces among heterosexual individuals (van Hooff et al., 2011). Nevertheless, although some researchers have found attentional bias toward HA male faces among female observers, they also indicate that such an effect is much weaker than the attentional bias toward HA female faces among male observers (van Straaten et al., 2010; Valuch et al., 2015). The difference between the hypotheses of “female beauty captures attention” and “opposite-sex beauty captures attention” requires further investigation.

Regarding infant faces, evolutionary theory proposes that facial attractiveness or cuteness is a powerful protective mechanism that is beneficial for the survival of infants, through capturing attention, enhancing nurturing intention, eliciting positive affect and protective behaviors, and reducing the possibility of aggression against infants (Borgi et al., 2014). Furthermore, the cuteness of infants can also be an indicator of their health (Golle et al., 2015). According to parent-offspring conflict theory, parents may refrain from investing in newborns until the risk of infant death is reduced to maximize parental investment (Trivers, 1974). Therefore, a cute infant may acquire more nursing and caring compared to a less cute infant. In other words, observers may allocate more cognitive resources to HA infant faces than to LA infant faces. As the motivations are different for the processing of attractiveness in adult and infant faces, the difference in the findings concerning adult and infant faces can be interpreted from different perspectives. First, from the perspective of mate selection, the sex of the face is a determining factor in the processing of facial attractiveness. However, from the perspective of raising offspring, the sex of the face is not important, and instead, the sex of the observer is more crucial in raising offspring. Therefore, there is little evidence showing that the sex of infant faces influences the processing of facial attractiveness. Second, from the perspective of mate selection, men but not women emphasize the physical attractiveness of the mate. Therefore, the effects of facial attractiveness on cognitive processing are greater in male observers than in female observers (Kleck and Rubenstein, 1975; Fugita et al., 1977; van Straaten et al., 2010; Valuch et al., 2015; Mitrovic et al., 2018). However, from the perspective of raising offspring, women play a more important role than do men. Therefore, women are more sensitive to the attractiveness of infant faces than are men (Glocker et al., 2009a; Sprengelmeyer et al., 2009; Lobmaier et al., 2010). Finally, from the perspective of mate selection, romantic relationship status is a key factor in deciding whether to seek a mate or not. Therefore, single individuals show stronger cognitive bias toward HA adult faces compared with non-single individuals (Karremans et al., 2011; Mitrovic et al., 2016). However, from the perspective of raising offspring, the hormone levels of women are crucial. Therefore, women with a high hormone level are more sensitive to the attractiveness of infant faces than are women with a low hormone level (Sprengelmeyer et al., 2009).

In summary, in the theoretic framework of evolutionary psychology, a variety of findings concerning facial attractiveness in both adult and infant faces could be explained through reproductive motivations. However, it should be noticed that the evolutionary psychology is not the only theory which could be used to explain experimental findings. Other factors may also influence the cognitive processing of facial attractiveness. For example, the evidence that women showed attentional bias not only to HA male faces but also to HA female faces might potentially be explained from the perspectives that women might be more flexible in sexual orientation or more sensitive in judging the same gender’s attractiveness. However, as the current review mainly concerns the evolutionary perspective, these possibilities were not emphasized here and might be discussed in further studies.

## Summary and Prospects

To summarize, there are common and different mechanisms underlying the cognitive processing of facial attractiveness in adult and infant faces. On the one hand, there are similarities between the processing of facial attractiveness in adult and infant faces. That is, humans generally show cognitive biases to more attractive faces for both adults and infants, which demonstrates the evolutionary significance of facial attractiveness. On the other hand, different factors may determine the cognitive processing of the adult and infant faces. For example, the sex of the face, from the perspective of “female beauty captures attention,” is the determinant factor of the processing of adult faces, while the sex of the observer is the determinant factor of the processing of infant faces. The perspectives of mate selection and raising offspring in evolutionary psychology are proposed as potential mechanisms for the processing of facial attractiveness in adult faces and infant faces, respectively. More studies are needed to further elucidate the mechanisms underlying the processing of facial attractiveness.

First, facial cuteness is usually considered the main characteristic of infant facial attractiveness. However, cuteness can also be a characteristic of an adult face. In adult faces, cuteness and attractiveness seem to be two independent characteristics in Western cultures (Geldart, 2010). However, in Eastern cultures such as Japan, a cute female face is considered attractive. Investigations in Japan suggest that cuteness indicates non-sexual attractiveness in female faces for women while it indicates both sexual and non-sexual attractiveness in female faces for men (Cole et al., 2015). Neurophysiological evidence showed that cute adult faces were related to activations in broad regions such as the bilateral insula, bilateral anterior cingulate cortex and right inferior frontal gyrus among Japanese men (Wiese E. et al., 2014). However, whether the mechanisms for the cognitive processing of cuteness are the same as those for the processing of attractiveness in adult faces remains unknown. Therefore, comparisons of these mechanisms between Eastern and Western cultures are an interesting investigation direction.

Second, most findings can be interpreted by evolutionary theory, which emphasizes reproductive motivation in the processing of facial attractiveness. However, under some circumstances, reproduction motivation is relatively weak. Sex orientation is one of the most important factors which may affect the processing of facial attractiveness in adult faces. For example, an eye-movement tracking study revealed that both heterosexual women and homosexual men showed longer total fixation duration and more frequent fixations at HA male faces which were consistent to their sex orientation (Mitrovic et al., 2016). Neurophysiological evidence showed that activities in OFC were higher for HA male faces than HA female faces in heterosexual women and homosexual men, whereas the activities were higher for HA female faces than HA male faces in heterosexual men and homosexual women (Manera et al., 2014). Therefore, other theories should be developed to explain these findings. For example, Sato et al. (2007) proposed a tripartite model of neuroaesthetics which emphasizes cognitive neuroscience framework rather than evolutionary framework. According to this model, aesthetic experiences might emerge from the interaction between emotion–valuation (including reward, emotion and wanting/liking), sensory–motor (involving sensation, perception and motor system), and meaning–knowledge (including expertise, context and culture) neural systems. The stimuli of HA faces may activate these systems in individuals who prefer these stimuli. In addition to the neuroaesthetic model, cognitive behavior model might be another framework to explain the results regarding facial attractiveness. According to the cognitive behavior model of body image disturbance, the cognitive processing for schema-consistent stimuli is facilitated among individuals with maladaptive body self-schemata (Chaminade and Okka, 2013). Accordingly, Kou et al. (2016) found that women with dissatisfaction for their facial attractiveness showed attentional bias toward LA female faces. Other theories besides the evolutionary theory should be further investigated.

Third, in addition to the face, other cues can express one’s attractiveness, such as social status, personality, body attractiveness, and voice attractiveness. Some cues are closely related to facial attractiveness and thus may share similar mechanisms. For example, for adults, women’s facial attractiveness has been found to be related to body shape (i.e., waist-hip ratio), while men’s facial attractiveness has been found to be related to income (Shin et al., 2018). An eye-movement tracking study showed that both men and women spend more time viewing high status male targets than high status female targets (Dewall and Maner, 2008). For infants, it is proposed that positive infant sound and smell may contribute to the cuteness of infants (Kremláček et al., 2016). For example, the syllabic sound produced by 3-month-old boys was rated cuter than that produced by girls, even the boys were labeled with girls’ names (Nuku and Bekkering, 2008). Further studies are needed to elucidate the mechanisms underlying the processing of these cues and to test evolutionary theory in these studies. In addition, the integration and interaction among different attractiveness cues are also worthy of investigation.

## Author Contributions

HK participated in writing the manuscript. QX participated in data collection. TB participated in study design and revising the manuscript critically. All authors read and approved the final manuscript.

## Conflict of Interest

The authors declare that the research was conducted in the absence of any commercial or financial relationships that could be construed as a potential conflict of interest.
